# High Efficacy of Ozonated Oils on the Removal of Biofilms Produced by Methicillin-Resistant *Staphylococcus aureus* (MRSA) from Infected Diabetic Foot Ulcers

**DOI:** 10.3390/molecules25163601

**Published:** 2020-08-07

**Authors:** Vanessa Silva, Cecília Peirone, Joana S. Amaral, Rosa Capita, Carlos Alonso-Calleja, José A. Marques-Magallanes, Ângela Martins, Águeda Carvalho, Luís Maltez, José Eduardo Pereira, José Luís Capelo, Gilberto Igrejas, Patrícia Poeta

**Affiliations:** 1 Microbiology and Antibiotic Resistance Team (MicroART), Department of Veterinary Sciences, University of Trás-os-Montes and Alto Douro (UTAD), 5000-801 Vila Real, Portugal; vanessasilva@utad.pt; 2 Department of Genetics and Biotechnology, University of Trás-os-Montes and Alto Douro, 5000-801 Vila Real, Portugal; gigrejas@utad.pt; 3 Functional Genomics and Proteomics Unit, University of Trás-os-Montes and Alto Douro (UTAD), 5000-801 Vila Real, Portugal; 4 Associated Laboratory for Green Chemistry (LAQV-REQUIMTE), University NOVA of Lisboa, Lisboa, 2829-516 Caparica, Portugal; 5 Department of Veterinary Sciences, University of Trás-os-Montes and Alto Douro (UTAD), 5000-801 Vila Real, Portugal; cecilia_peirone@yahoo.com.ar (C.P.); lmaltez@utad.pt (L.M.); jeduardo@utad.pt (J.E.P.); 6 Hospital Center of Trás-os-Montes and Alto Douro E.P.E., 5000-185 Vila Real, Portugal; aguedasenacarvalho@gmail.com; 7 Centre for the Research and Technology of Agro-Environmental and Biological Sciences (CITAB), UTAD, 5000-801 Vila Real, Portugal; 8 Centro de Investigação de Montanha (CIMO), Instituto Politécnico de Bragança, 5300-253 Bragança, Portugal; jamaral@ipb.pt; 9 REQUIMTE-LAQV, Department of Chemical Sciences, Pharmacy Faculty, University of Porto, 4050-313 Porto, Portugal; 10 Department of Food Hygiene and Technology, Veterinary Faculty, University of León, E-24071 León, Spain; rosa.capita@unileon.es (R.C.); carlos.alonso.calleja@unileon.es (C.A.-C.); 11 Institute of Food Science and Technology, University of León, 24004 León, Spain; 12 Critical Care Department, University Hospital Braga, 4710-243 Braga, Portugal; jose.regojo@yahoo.com; 13 Department of Zootechnics, University of Trás-os-Montes and Alto Douro (UTAD), 5000-801 Vila Real, Portugal; angela@utad.pt; 14 Veterinary and Animal Research Centre, University of Trás-os-Montes and Alto Douro (UTAD), 5000-801 Vila Real, Portugal; 15 BIOSCOPE Group, LAQV@REQUIMTE, Chemistry Department, Faculty of Science and Technology, NOVA University of Lisbon, 2825-466 Almada, Portugal; jlcm@fct.unl.pt; 16 Proteomass Scientific Society, 2825-466 Costa de Caparica, Portugal

**Keywords:** ozone, ozonated oil, MRSA, *S. aureus*, biofilms, diabetic foot ulcers

## Abstract

Ozone has a high wound healing capacity and antibacterial properties and can be used as a complementary treatment in infections. Methicillin-resistant *S. aureus* (MRSA) is the most common pathogen found in infected diabetic foot ulcers. Most of MRSA are resistant to several classes of antibiotics and, therefore, there is a need for new, effective, and well-tolerated agents. Thus, we aimed evaluate the antimicrobial and antibiofilm potentials of ozonated vegetable oils against MRSA strains isolated from diabetic foot ulcers. Six ozonated oils were produced with concentrations of ozone ranging from 0.53 to 17 mg of ozone/g of oil. The peroxide values were determined for each oil. Ozonated oils content on fatty acid was determined by gas chromatography equipped with a flame ionization detector. The antimicrobial susceptibility testing was performed by the Kirby–Bauer disk diffusion method and the effect of ozonated oils on biofilm formation ability and on established biofilms was investigated. In general, the content in identified unsaturated fatty acid in oils decreased with the increase of ozonation time and, consequently, the peroxide value increased. Most bacterial strains were inhibited by ozonated oil at a concentration of 4.24 mg/g. Ozonated oils had moderate to high ability to remove adhered cells and showed a high capacity to eradicate 24 h old biofilms. Our results show promising use of ozonated oils on the treatment of infections, in particular those caused by multidrug-resistant MRSA strains.

## 1. Introduction

Ozone is an inorganic and powerful oxidant compound that consists of three oxygen atoms. It is an essential gas for life on Earth, being produced naturally in the Earth’s atmosphere [1,2]. Its first known medical application was the purification of blood in test tubes in 1870 [1]. Ozone reacts with several biological structures that are indispensable to life. However, ozone does not have a cytotoxic effect; on the contrary, it induces fibroblast migration aiding the wound-healing process [3,4]. Over the years, ozone has been used as a treatment option for several conditions such as diabetic foot ulcers (DFUs), periodontal disease, and chronic inflammation, among others [5]. Moreover, ozone has also been used as a disinfectant in wastewater treatment and drinking water preparation owing to its high antimicrobial activity [6]. Indeed, several studies have shown that, because of its non-specific action, ozone has an effect on bacteria, virus, protozoa, and fungi [4]. One of the first published studies on the use of ozone as a disinfectant and bactericide was performed by a military doctor in 1916, who reported the disappearance of pus from the treated wounds and their rapid healing [7]. Ozone blocks the enzymatic function on bacteria by oxidizing glycoproteins and glycolipids. To enter inside the bacterial cell, ozone oxidizes the phospholipids and lipoproteins of the bacterial cell envelope, disrupting the cytosolic membrane integrity [8,9]. Owing to its high wound healing capacity, its influence on the processes of oxygen metabolism, and antibacterial properties, ozone has been used as a complementary treatment in infected DFU, impairing the healing and reducing the infection and amputation rates [10]. DFUs are one of the main causes of morbidity in diabetic patients and it is estimated that half of the DFUs will develop an infection [11]. The treatment provided for infected DFU is often inadequate, resulting in preventable complications and prolonged and unnecessary healing times. DFUs are typically infected by several different microorganisms; however, *Staphylococcus aureus* are known to be the most prevalent, playing a significant role in diabetic foot infections [12]. Methicillin-resistant *S. aureus* (MRSA) also represents a serious threat, as 30 to 50% of DFU are colonized by MRSA strains [12]. In fact, in a previous study, we reported a high prevalence (93.8%) of *S. aureus* in infected DFU, of which 64.1% were methicillin-resistant. Furthermore, the majority of MRSA strains belonged to epidemic clones [11]. *S. aureus* strains are frequently found on the skin and in the nasal mucosa of about 30% of healthy humans living in relationship of commensalism or mutualism with the host [13]. However, *S. aureus* are also responsible for different types of infections, including skin infections, endocarditis, osteomyelitis, or septicemia [14]. MRSA are often implicated with increased virulence and resistance to multiple antibiotic classes, which makes infections caused by MRSA extremely difficult to treat, particularly chronic infected wounds, as 80% of all human chronic infections are the result of bacterial biofilms [15]. Biofilm formation is a complex multi-step process that is often associated with several bacterial species [16]. However, biofilm arising on the surface of medical materials or human tissues is mainly formed by one bacterial species. Biofilm formation comprises several stages, namely, the initial attachment to the surface, micro-colony formation, maturation, and detachment/dispersion of biofilm [15]. Adhesion to the surfaces is a fundamental step in biofilm formation, which leads to significant changes in cell metabolism, mainly to the expression of extracellular polymeric substances (EPS) and various microbial surface components, which recognize adhesive matrix molecules (MSCRAMMs), which have affinity, among others, to fibronectin and fibrinogen [17]. Once established, MRSA biofilms are refractory to antimicrobial and immune host response, becoming almost impossible to eradicate [18]. Moreover, MRSA biofilms formed on DFUs develop protected microenvironments, which shield bacteria from the action of neutrophils, increasing their resistance to antibiotics [19]. Furthermore, under these conditions, when the biofilm is formed by multidrug-resistant MRSA strains, there is an enhancing of the overall resistance. Therefore, as the prevalence of biofilm-infected wounds increased in the last few years and the current strategies fail to suppress biofilm infections, there is an urgent need for effective therapies [20]. Thus, this study aims to chemically characterize ozonated oils with different concentrations and to evaluate the ability of ozonated oils to control pre-established adhered cells and 24 h old biofilms of multidrug-resistant MRSA and methicillin-susceptible *S. aureus* (MSSA) strains isolated from infected DFUs.

## 2. Results

### 2.1. Determination of Peroxide Value

Six ozonated vegetable oils were produced with concentrations of ozone 0.53, 1.06, 2.12, 4.24, 8.48, and 17 mg/g of oil corresponding to different exposure periods of the gas stream: 10, 20, 40, 80, 160, and 320 min, respectively. The determination of peroxide value was performed by iodometric titration and the results are shown in Table 1. A lower peroxide value was detected in control sample (CTR) when compared with sample 1 (44.4 ± 3.4 meq O_2_/kg), which corresponds to the lowest ozonation time (10 min). The peroxide value increased an average of 1.25-fold between samples with the increase of ozonation time until sample 5 (160 min); however, between samples 5 and 6, which had a peroxide value of 113.5 and 220.7 meq O_2_/kg, respectively, there was a much more significant increase of 1.9-fold.

### 2.2. Fatty Acid Content Analysis

Fatty acid methyl esters (FAMEs) present in our oil samples were analyzed by gas chromatography-flame ionization detector (GC-FID), and the results are expressed in Table 2. Samples 1, 2, 3, 4, 5, and 6 correspond to 10, 20, 40, 80, 160, and 320 min of ozone treatment, respectively. In control samples, it was possible to identify all fatty acids, whereas it was possible to identify 97.99% of the fatty acids contained in sample 1. A high proportion (87.10%) of unsaturated fatty acids and a low proportion (12.91%) of saturated fatty acids were detected in the mixture of olive and sunflower oil. Palmitic acid (C16:0) was the major saturated fatty acid found in this study, representing 8.45% and 14.67% of total fatty acids in the control sample and in sample 6, respectively. The main fatty acids present in the oil mixture were the oleic (C18:1n9c) and linoleic acids (C18:2n6c), which account for about 82% of the identified fatty acids in sample 1 (10 min). Overall, the percentage of identified fatty acids decreased progressively with the increase of ozonation time as well as with the increase of the peroxide values (Table S1); however, the percentages of identified unsaturated fatty acids in samples treated for 160 and 320 min decreased rapidly from 82.76% to 58.00% and, consequently, there was also a significant drop in the percentage of oleic and linoleic acids as a result of 360 min of ozonation. On the contrary, the oil content on almost all saturated fatty acids increased significantly with the ozonation time. The unsaturated fatty acids, with the exception of α-linolenic acid (C18:3n3), were identified in both treated and control samples.

### 2.3. Antimicrobial and Antibiofilm Activities

**Figure 1:**
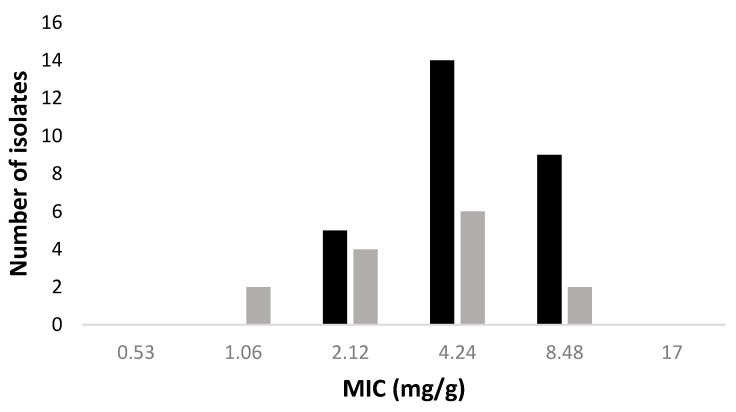
Minimum inhibitory concentrations (MIC) of ozonated oils against 28 methicillin-resistant *S. aureus* (MRSA) (black) and 10 methicillin-susceptible *S. aureus* (MSSA) (grey) strains.

**Figure 2:**
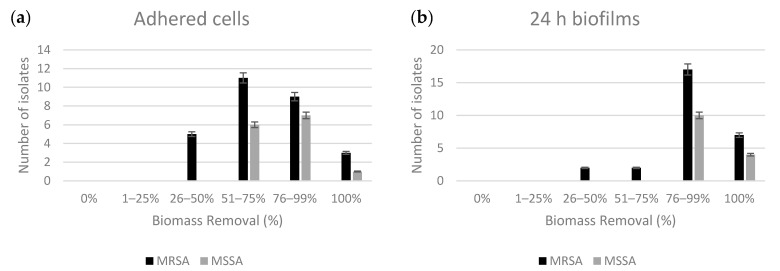
Percentage of biomass removal of ozonated oils at MIC value on (**a**) adhered cells and (**b**) 24 h old biofilms of 28 MRSA (black) and 10 MSSA (grey) strains.

The antimicrobial susceptibility testing was preformed against 28 MRSA and 14 MSSA strains isolated from infected DFUs. Most MRSA and MSSA strains were inhibited by sample 4 (80 min of ozonation), which has an ozone concentration of around 4.24 mg/g of oil. The minimum inhibitory concentration (MIC) results are shown in Figure 1. Oils containing lower concentrations of ozone (1.06 and 2.12 mg/g) were also able to inhibit the growth of two MSSA and five MRSA, respectively. All MRSA and MSSA strains were susceptible to the ozonated oils, with MSSA strains being more susceptible than MRSA. Furthermore, the majority of strains presented a high inhibition zone of 15 to 17 mm when exposed to high concentrations of ozone (8.48 and 17 mg/g). The effect of ozonated oils on adhered cells and established biofilms was also investigated. For both assays, the ozonated oils at MIC value were used and the percentage of biomass removal was calculated. Ozonated oils had moderate to good ability to remove adhered cells (Figure 2). Ozonated oil at MIC value removed 51 to 75% and 76 to 99% of biofilm mass on 11 of the 28 MRSA and 7 out of 14 MSSA strains, respectively. Moreover, the biomass of three MRSA and one MSSA was completed removed. Regarding efficacy of ozonated oils on 24 h old biofilms, the biomass removal of all MSSA strains was superior to 75%. The 24 h old biofilms of 17 MRSA strains suffered a biomass removal of between 76 and 99%.

## 3. Discussion

In our experiment, six ozonated oils were produced with different concentrations of ozone. As expected, it was possible to observe that, as the ozonation time increased, the oil mixture gradually loses its original yellow color and eventually gains a colorless appearance [21]. Furthermore, as also expected, with increasing ozonation time, the oil exponentially becomes more viscous, which was visually checked in this study. It has been reported that ozonated oil viscosity can reach values up to 984 mPa [22]. These changes in the physico-chemical characteristics of the ozonated oils occur owing to several reactions that take place during ozonation, leading to the formation of polymeric peroxides, which increase the viscosity, as well as reactions of hydrolysis of the formed oxygenated products, which increase the acidity [22]. Furthermore, the increased viscosity of the oils observed after ozonation is owing to the dimension and orientation of molecules, that is, the decrease in the degree of unsaturation as well as the increase of the molar mass promote the increase in viscosity [23]. The peroxide value represents an essential measurement to establish the therapeutic dose in vegetable oils as it measures the quantity of the main products of vegetal oil ozonation, which are trioxolanes and peroxides. These species are responsible for the therapeutic and biological effects of ozonated oils [24]. As expected, there was an increase in the peroxide value with increasing ozonation time, with the peroxide value of sample 6 (320 min of treatment) being about 56 times higher than the control sample. Indeed, sample 6 was completely colorless and almost solid. Pai et al. (2014) evaluated the wound healing capacity of ozonated sesame oil with higher and lower peroxide values (500 and 700 meq O_2_/kg oil) and reported that the high dose had a better effect on the acceleration of wound healing [25].

Olive and sunflower oils are formed mainly by triglycerides; however, their composition in sutured and unsaturated fatty acids varies with their origin and nature [26,27]. In our study, and as previously reported, the major saturated fatty acid olive oil is palmitic acid [28,29]. Other studies have reported that those two fatty acids are the predominant ones in sunflower oil, representing nearly 90% of the fatty acid content [30,31]. Oleic and linoleic acids are also the main fatty acids in olive oil, representing between 55 and 83% and 3.5 and 21% of the total fat, respectively [28]. From the C18 series, oleic and linoleic acids, with one and two double bonds, respectively, along with linolenic acid (three double bonds), are the most important fatty acids in vegetable oils [21]. The α-linolenic acid (C18:3n3) was also detected in our study; however, in a much smaller proportion than the other two C18 acids. Besides, the health benefits of olive oil are also thanks to the presence of other minor compounds, such as polyphenols, tocopherols, carotenoids, and sterols [28]. In general, the content of identified unsaturated fatty acid in oil decreased with the increase of ozonation time and, consequently, the peroxide value increased. On the contrary, almost all saturated fatty acids, except C20:0, increased significantly with the time of ozonation, reaching its highest value in sample 6 (360 min of ozonation), which is consistent with other studies, because oxidation causes the increase in relative percentages of saturated fatty acids and a decrease of relative percentages of unsaturated fatty acids [32,33]. All unsaturated fatty acids, except the α-linolenic acid (C18:3n3), were detected in all samples, which indicates that ozone did not react with all double bonds of these acids, which remained as modified triglycerides and non-modified triglycerides, and can then be oxidized by the oxygenated compounds formed [31]. The percentages of identified unsaturated fatty acids in samples 5 and 6 (160 and 320 min of treatment) decreased rapidly, which is in accordance with the results obtained in other studies, leading to a decrease in the percentages of oleic and linoleic acids [32,34]. The oxidation of oleic acid comprises the break of the carbon–carbon double bond at position nine of the carbon backbone [35]. Moreover, the reaction of ozone with oleic acid generates oxidation products, commonly called Criegee intermediates, which are highly reactive and can undergo the formation of ester and hydroperoxide [35]. The reaction of ozone with vegetable oils occurs exclusively within the carbon–carbon double bonds of the unsaturated fatty acid. In fact, Sega et al. (2010) analyzed ozonated vegetal oils by ^1^H-NMR spectroscopy and confirmed that the reaction of ozone with the double bond leads to its gradual disappearance [22]. The main product formed during ozonation is the 1,2,4-trioxolane; nevertheless, other oxygenated compounds such as ozonides, peroxides, hydroperoxides, aldehydes, deperoxides, and polyperoxides are also produced. Those oxygenated compounds can also account for the wide biological activity of ozonized oils, including the antimicrobial activity [36].

*S. aureus* infections are particularly difficult to treat because most *S. aureus* easily acquire antimicrobial resistance gene and, consequently, *S. aureus* found in a hospital environment are usually multi-drug resistant. All ozonated oils were tested against 28 MRSA and 14 MSSA isolates from infected DFUs. Overall, the ozonated oils showed excellent results against the tested strains. It is also important to point out that all strains used in this study were susceptible to the ozonated oils, with MSSA strains being more susceptible to ozonated oils than MRSA. The antimicrobial effect of ozonated oils may varies according to the type of oil used, as the number of carbon–carbon double bonds can be different from one oil to another; therefore, during the ozonation, the amount of unsaturation could impact the antimicrobial activity of ozonated oils [37]. A study has shown that ozonated sunflower oils have a broad antibacterial spectrum, exhibiting an inhibitory effect not only on Gram-positive bacteria, but also on Gram-negative, including antimicrobial resistant strains [38]. Other studies have been conducted to evaluate the efficacy of ozone against *S. aureus*. Sechi et al. (2011) and Rodrigues et al. (2004) used the ozonised oils, Oleozon^®^ (Centro de Investigaciones del Ozone, Havana, Cuba) and Bioperoxoil^®^ (Ozonoil, Barcelona, Spain), respectively, and reported MIC values of 9.5 mg/mL for *S. aureus* [38,39]. In our study, all strains were inhibited at lower concentrations. This difference in results could be attributed to different matrixes used, to different bacterial strains, and to different peroxide values. In fact, the peroxide values of ozonated oils of these studies were fairly higher than ours, ranging from 500 to 800 mmol/kg, with the best results for antimicrobial activity being achieved with oils showing peroxide values of 650 mmol/kg, whereas in our study, the best MIC results were obtained at 101.7 ± 4.7 meq O_2_/kg. Skalska et al. (2009) investigated the antibacterial activity on Gram positive and negative strains of ozonated sunflower oils, and reported MICs of 200 and 250 mg/mL of ozone, respectively, which are much higher concentrations [40]. The use of high doses of ozone may raise some questions regarding toxic effects. However, studies have shown that ozone has a biocompatibility with human gingival, fibroblast, and periodontal cells [41,42]. As mentioned before, the marked action on antibacterial activity of ozonated oils has been attributed to several ozonated oil components, in particular, to trioxolanes and peroxides, which are the main chemical products from oil ozonolysis [31]. The mechanisms of action of these products are owing to their action on the destruction of bacterial cell walls and the cytoplasmic membrane [43]. The ozonated oil affects bacterial cell permeability, induced by the loss of intracellular K^+^ ion content, and leading to a reduction of cytoplasmic content in *S. aureus* bacterial cells. Moreover, the effect of ozone on bacteria cell membrane may also be the result of the high instability of ozone that leads to its rapid decomposition in free radicals, which spread quickly through the bacterial cell, disturbing the usual cellular activity [44,45]. Nevertheless, bacteria prefer to grow as communities called biofilms, which are responsible for nearly 80% of all human infections, and one of their most critical features is their considerably higher resistance to environmental stresses, antimicrobials, disinfectants, and host immune defenses [46]. Even so, most antimicrobial resistance research has been focused on bacteria growing in planktonic cultures and antimicrobials were originally developed to target individual bacterial cells [47]. Indeed, although several studies have reported the antibacterial effect of ozonated oils, only a few studies have investigated their anti-biofilm potential on MRSA strains. In this study, we also evaluated the capacity of ozonated oils to control adhered cells and its effect on established biofilms. Overall, ozonated oils presented a moderate to good ability to remove adhered cells and a good ability to remove the biofilm biomass of 24 h biofilms. Nevertheless, ozonated oils seemed to have a higher efficacy against MSSA than MRSA strains. This may be owing to the fact that acquiring methicillin resistance probably suppresses production polysaccharide intercellular adhesin (PIA)-dependent biofilm and promotes the formation of surface-associated biofilms because the presence of *mec*A gene (responsible for methicillin resistance) inhibits PIA-dependent biofilm, whereas MSSA commonly produce a PIA-dependent biofilm [47]. Furthermore, the presence of virulence factors may also play an important role in *S. aureus* biofilm formation, because both biofilm formation and virulence factor secretion are mediated by accessory gene regulator, staphylococcal accessory element, and staphylococcal accessory regulator A [1]. However, there seems to be a difference between MRSA and MSSA as the deletion in the *agr* gene expression regulation system, for instance, affects the increase of biofilm formation in MRSA strains, while not having a significant effect on MSSA strains [2]. Ozone appears to be an alternative to conventional antimicrobials and disinfectants. A study conducted with a chronic wound infection model to investigate the efficacy of wound care products against *Staphylococcus* spp. reported that ozonated olive oil had by far the highest activity against biofilms [48]. Not only ozonated oils, but also ozonated water proved to be effective on biofilm removal of *S. aureus*. Bialoszewski et al. (2011) studied the antimicrobial activity of ozonated water against *S. aureus* and *Pseudomonas aeruginosa* biofilms. The biofilms were allowed to grow for 24, 48, and 72 h and the freshly ozonated water showed a high efficacy on the removal of both *S. aureus* and *P. aeruginsa* biofilms [49]. Another study conducted with ozonated water has also shown its efficacy on the removal of biofilm of MRSA [50]. The efficacy of ozone to remove *S. aureus* biofilms from metal surfaces was evaluated and, after 360 min of ozone treatment, no viable biofilm bacterial cells were detected, which shows the promising use of ozone on the treatment of implant-related infections [51]. DFUs and other chronic wounds represent an ideal environment for biofilm development because, owing to impaired immune response, wounds are susceptible to infection and the presence of necrotic tissue promotes cell attachment. Biofilms in DFUs affect the inflammatory cellular response and function, the wound healing, and the cutaneous innate immune response. Furthermore, although biofilm infections are not eradicated by the host immune system, they also show resistance to systemic and topical antibiotics, which is believed to be because of slow diffusion of antimicrobials and intrinsic features of biofilm bacteria [52]. Therefore, ozone may be a good alternative to antibiotics to treat chronic wounds biofilms, particularly DFU. Besides the recognized bactericidal effect of ozone, studies have shown that programmed cell death may occur within biofilm communities as part of a defense mechanism owing to stressful conditions such as high temperatures, oxidative stress, amino-acid starvation, radiation exposure, and antimicrobial treatments [53]. Nevertheless, besides the antibacterial and antibiofilm properties of ozonated oils, it should be noted that ozone reacts immediately with the double bonds of the fatty conferencing the ozonated oils properties similar to ozone gas, but with greater physical and chemical stability, remaining stable for 2 years at 4 °C [34]. Furthermore, ozone application has been accepted as one of the main approaches for sustainable and clean technologies development, as no by-products are produced during ozone-based processes [54].

## 4. Material and Methods

### 4.1. Oil Ozonisation Procedure

Ozonated oils were prepared from a 50:50 mixture of extra virgin olive oil and refined sunflower oil. Briefly, 100 mL of the mixture was subjected to a gas stream of O_2_/O_3_ mixture at a concentration of 75 µg/mL of ozone, in continuous flow of 4 L/min and under normal pressure conditions. Ozone was generated by passing oxygen through a HYpermedozon generator (Herrmann, Elsenfeld, Germany). Six ozonated oils were produced with concentrations of ozone of 0.53, 1.06, 2.12, 4.24, 8.48, and 17 mg/g of oil corresponding to different exposure periods of the gas stream: 10, 20, 40, 80, 160, and 320 min, respectively.

### 4.2. Determination of Peroxide Value

Peroxide value was determined according to the Portuguese Standard NP-904:1987. Briefly, 0.5 g of oil was added to 15 mL of glacial acetic acid, 10 mL chloroform, and 1 mL potassium iodide saturated solution. After agitation, the sample was left 5 min in the dark and then the liberated iodine was titrated with sodium thiosulfate 0.01N, using a starch indicator. Peroxide value was expressed as milli-equivalents (meq) peroxide per 1 kg oil.

### 4.3. Fatty Acid Methyl Esters Analysis

Fatty acids of ozonized sunflower and olive oils samples were analyzed. For the gas chromatography analysis, the methyl ester derivatives were first prepared. Briefly, 100 µL of butylated hydroxytoluene (BHT) (Merck KGaA, Darmstadt, Germany) (0.01% in hexane) and 1.25 mL of KOH (0.25M in methanol) were added to 12.5 mg of oil and homogenize by vortex mixing. The mixture was heated for 10 min at 100 °C and the solution was allowed to cool down completely. One millilitre of BF_3_ solution (14% in methanol) was added, and the mixture was homogenized by vortex mixing and heat for 30 min at 100 °C. After the solution completely cooled down, 2 mL of n-hexane was added, and the solution was homogenized by vortex mixing. Then, 1 mL of saturated NaCl solution was added, and the mixture was homogenized and then centrifugated for 5 min at 3000 rpm. Finally, the upper layer was collected in a new vial and Na_2_SO_4_ anhydrous was added.

Fatty acid methyl esters were analysed using a Bruker^®^ SCION 436-GC gas chromatograph (Bruker, Billerica, MA, USA) equipped with a flame ionization detector (GC-FID), a split–splitless injector, and a Bruker CP-8410 autosampler. The FID temperature was set at 270 °C with a sampling frequency of 50 Hz, the injector temperature was set at 260 °C with a split ratio of 1:25, and the autosampler programmed to inject 1 μL. Compounds were separated on a fused carbon–silica column, coated with a cyanopropyl phase (CP-Sil 88, 50 m × 0.25 mm i.d and 0.20 μm film thickness, Agilent J&W (Palo Alto, CA, USA, country). The oven temperature was set at 160 °C and held for 3 min, increase at 3 °C/min to 229 °C, and held for 2 min. Helium was used as a carrier gas with a column flow of 1 mL/min. Data acquisition and processing were performed using the CompassCDS 3.0 (Bruker, Germany) software for GC systems. The peaks on the samples corresponding to FAME were identified by comparison of retention time with the Certified Reference Material of 37 component FAME mix from Supelco (Sigma Aldrich, St. Louis, MO, USA). Fatty acids were identified as FAME and quantified based on the obtained peak area, with the exception of C18:1n7, which is not present on the 37 FAME mix and was identified based on data from the literature [55,56]. The results were expressed as relative percentages considering all the peaks in the samples, including unknowns.

### 4.4. Bacterial Isolates

Antimicrobial susceptibility testing was performed against 28 MRSA and 14 MSSA strains isolated from infected DFU. The antibiotic resistance, virulence, and genetic lineages of MRSA isolates were previously characterized [11,57]. Of these 28 MRSA isolates, 20 were classified as multidrug-resistant, with the great majority being associated with two epidemic clones, namely EMRSA-15 (57%) and New York Japan (or related) (21%). The strains are part of the University of Trás-os-Montes and Alto Douro collection. All bacterial strains were grown in Brain Heart Infusion (BHI) agar (Oxoid, Basingstoke, UK) for 24h at 37 °C. For the antimicrobial activity assay and biofilm formation assays, Müller–Hinton (Oxoid, Basingstoke, UK) agar and broth were used in the same previous conditions.

#### Antibacterial Susceptibility Test

Ozonized oils with ozone doses around 0.53, 1.06, 2.12, 4.24, 8.48, and 17 mg/g were used in microbiological studies. The antimicrobial susceptibility testing was performed by the Kirby–Bauer disc diffusion assay. Bacteria were seeded on BHI agar and grown at 37 °C for 24 h. Then, 20 µL of each oil sample was loaded on sterile blank discs (6 mm diameter) and allowed to settle for a few hours in the dark. A bacterial suspension with a turbidity equivalent to 0.5 McFarland standard was prepared for each bacterium and the inoculum was seeded onto Müller–Hinton agar. The discs impregnated with ozonated oil were placed onto the inoculated agar. Discs with antibiotics were used as positive controls and discs impregnated with the mixture of extra virgin olive oil and refined sunflower oil were used as negative control. The plates were incubated for 18 to 24 h at 37 °C. The inhibition zones, indicating the antimicrobial activity of ozonated oils, were measured with a ruler. The minimum inhibitory concentration (MIC) was established as the minimum concentration used, in which an inhibition zone (>7 mm) was visible.

### 4.5. Antibiofilm Effect of Ozonated Oils Using Crystal Violet (CV) Assay

#### 4.5.1. Effect on Biofilm Formation Ability

Ozonated oils at the MIC value were evaluated for their potential to inhibit cell attachment. Briefly, 96-well polystyrene microtiter plates were filled with 200 µL of bacterial suspension (OD_600nm_ of 0.04 ± 0.02) and incubated at 37 °C for 2 h without shaking to allow bacterial cells to attach to the surface of the plates. After incubation, the content of the plates was discarded, and the plates were rinsed three times in saline solution 0.9% (*w/v*) to remove non-adherent cells. Then, 180 µL of fresh medium and 20 µL of ozonated oils at MIC value were applied on adhered cells. An equal volume of oil mixture without treatment was added as negative control. After 24 h of incubation at 37 °C, the content of the plates was removed, the wells were washed with saline solution, and the plates were air-dried overnight.

#### 4.5.2. Effect on Established Biofilms

Biofilms were produced in 96-well microtiter plates. Briefly, 200 µL of bacterial suspension (OD_600nm_ of 0.04 ± 0.02) was added to each well and the plates were incubated at 37 °C for 24 h with shaking at 150 rpm. After the incubation period, the content of each well was removed and washed with saline solution 0.9% (*w/v*). Then, 20 µL of ozonated oil and 180 µL of fresh Muller–Hinton medium were added to 24 h old biofilms. Controls wells included oil mixture without treatment. The plates were again incubated at 37 °C for 24 h and 150 rpm. After the incubation, the content of each well was removed, washed three times with saline solution, and air-dried overnight.

#### 4.5.3. Assessment of Biofilm Biomass

To assess the biofilm mass, the attached bacteria were fixed with 200 µL of 96% (*v/v*) ethanol for 15 min. Then, the plate wells were emptied and 200 µL of 0.1% crystal violet was added to each well and allowed to stain for 10 min at room temperature. Afterwards, the excess of crystal violet was removed, and the plates were gently washed with saline solution and air-dried. Finally, 200 µL of 33% (*v/v*) glacial acetic acid was added to solubilize the crystal violet and the biomass was quantified by measuring the OD at 570 nm using a microplate reader BioTek ELx808U (BioTek, Winooski, VT, USA). The results are expressed as percentage of biomass reduction (%BR) in relation to biofilms non-exposed to ozonated oils:

(1) $$\%\mathrm{BR}=\frac{{\mathrm{OD}}_{\mathrm{CTR}}{-\mathrm{OD}}_{\mathrm{OT}}}{{\mathrm{OD}}_{\mathrm{CTR}}}\times 100$$

where OD_CTR_ is the OD_570nm_ value of control wells and OD_OT_ is the OD_570nm_ value for the ozone oil-treated wells.

### 4.6. Statistical Analysis

The results were analysed using one-way analysis of variance (ANOVA) followed by Tukey’s HSD test with *p* = 0.05. The results were expressed as mean values and standard deviation. Levene’s test was performed to evaluate the homogeneity of variances assumption needed for ANOVA. The analyses were carried out using IBM SPSS Statistics for Mac, Version 26.0. (IBM Corp., Armonk, NY, USA).

## 5. Conclusions

The ozonation of vegetable oils leads to a decrease in the amount of unsaturated fatty acids and an increase in peroxide values. Ozonated oils showed a high activity against both MSSA and MRSA strains isolated from DFUs showing an MIC value around 4.24 mg/g of oil. Furthermore, at this MIC value, ozonated oils had a moderate to high activity on the removal of adhered cells and 24 h old biofilms. Nevertheless, ozonated oils had a higher effect on the biomass removal of mature 24 h old biofilms than adhered cells, which is an advantage because DFUs quickly develop biofilm after infection. MRSA strains, of which most were multidrug-resistant, demonstrated higher resistance to ozonated oils than MSSA strains. The high capacity of ozonated oils to remove MRSA and MSSA biofilms, their action on planktonic bacterial cells, and their described safety features make ozonated vegetable oils a promising alternative to the current antibiotics and disinfectants. Our data clearly indicate that ozonated vegetable oils have an effect on bacterial biofilms, in particular in mature biofilms and adhered cells. Ozonated oils may be used as an alternative to antibiotics in the treatment of skin infections caused by multidrug-resistant bacteria. Further studies should be carried out regarding, for example, the application of ozonated oils on a DFU in vivo model.

## Figures and Tables

**Table 1:** Effect of ozone treatment on peroxide values. Values are presented as mean ± standard deviation (*n* = 2). CTR—control.

Sample	Ozonation time	Peroxide Value ^1^
CTR	0	3.9 ± 0.0 ^a^
1	10 min	44.4 ± 3.4 ^b^
2	20 min	69.5 ± 4.6 ^c^
3	40 min	83.4 ± 5.3 ^c^
4	80 min	101.7 ± 4.7 ^d^
5	160 min	113.5 ± 3.7 ^d^
6	320 min	220.7 ± 5.6 ^e^

^1^ Peroxide value was expressed as milli-equivalents (meq) peroxide per 1 kg oil. Analysis of variance (ANOVA) was performed, with different letters (a–e) indicating significant differences (*p* < 0.05).

**Table 2:** Percentage composition of fatty acids in oils control sample (CTR) and ozonated samples. Values are presented as mean ± standard deviation (*n* = 2).

Fatty acid	Sample CTR	Sample 1	Sample 2	Sample 3	Sample 4	Sample 5	Sample 6
**C14:0**	0.05 ± 0.00 ^a^	0.04 ± 0.00 ^a^	0.05 ± 0.00 ^a^	0.05 ± 0.00 ^a^	0.06 ± 0.00 ^a^	0.06 ± 0.00 ^a^	0.33 ± 0.02 ^b^
**C16:0**	8.45 ± 0.25 ^a^	8.65 ± 0.11 ^a^	8.91 ± 0.08 ^a^	9.23 ± 0.08 ^a,b^	9.94 ± 0.05 ^b^	11.53 ± 0.18 ^c^	14.67 ± 0.53 ^d^
**C16:1**	0.41 ±0.00 ^a^	0.42 ± 0.00 ^a^	0.40 ± 0.03 ^a^	0.06 ± 0.03 ^b,c^	0.06 ± 0.00 ^b,c^	0.04 ± 0.00 ^b^	0.12 ± 0.03 ^c^
**C17:0**	0.24 ±0,00 ^a^	0.24 ± 0.00 ^a^	0.41 ± 0.00 ^a,b^	0.78 ± 0.05 ^b^	1.57 ± 0.02 ^c^	2.72 ± 0.07 ^d^	6.58 ± 0.37 ^e^
**C17:1**	0.06 ±0.00 ^a^	0.06 ± 0.00 ^a^	0.06 ± 0.00 ^a^	0.05 ± 0.00 ^a^	0.03 ± 0.03 ^a^	0.05 ± 0.01 ^a^	0.65 ± 0.03 ^b^
**C18:0**	3.24 ± 0.14 ^a^	3.21 ± 0.00 ^a^	3.41 ± 0.01 ^a^	3.82 ± 0.08 ^a^	4.68 ± 0.02 ^b^	6.52 ± 0.17 ^c^	8.58 ± 0.40 ^d^
**C18:1n9c**	53.19 ± 0.68 ^a^	52.11 ± 0.68 ^a,b^	51.02 ± 0.07 ^b,c^	50.19 ± 0.38 ^c,d^	46.56 ± 0.16 ^e^	40.07 ± 0.51 ^f^	17.50 ± 0.60 ^g^
**C18:1n7**	2.29 ± 0.00 ^a^	2.18 ± 0.04 ^a,b^	2.16 ± 0.01 ^a,b^	2.07 ± 0.09 ^b,c^	1.98 ± 0.02 ^c^	1.76 ± 0.04 ^d^	0.49 ± 0.02 ^e^
**C18:2n6c**	30.70 ± 1.13 ^a^	29.72 ± 0.39 ^a^	28.89 ± 0.05 ^a,b^	27.35 ± 0.42 ^b^	23.24 ± 0.13 ^c^	17.05 ± 0.48 ^d^	5.87 ± 0.28 ^e^
**C18:3n3**	0.17 ± 0.02 ^a^	0.16 ± 0.01 ^a^	0.18 ± 0.01 ^a^	0.16 ± 0.02 ^a^	0.15 ± 0.01 ^a,b^	0.10 ± 0.02 ^b^	n.d.
**C20:0**	0.31 ± 0.01 ^a^	0.31 ± 0.02 ^a^	0.31 ± 0.00 ^a^	0.30 ± 0.03 ^a^	0.34 ± 0.07 ^a^	0.42 ± 0.03 ^a^	0.39 ± 0.02 ^a^
**C20:1**	0.27 ± 0.01 ^a^	0.27 ± 0.01 ^a^	0.27 ± 0.00 ^a^	0.24 ± 0.03 ^a,b^	0.19 ± 0.02 ^b,c^	0.12 ± 0.02 ^c^	0.14 ± 0.03 ^c^
**C22:0**	0.50 ± 0.05 ^a^	0.47 ± 0.08 ^a^	0.60 ± 0.01 ^a,b^	0.84 ± 0.08 ^b^	1.27 ± 0.01 ^c^	1.93 ± 0.09 ^d^	2.29 ± 0.13 ^e^
**C24:0**	0.13 ± 0.00 ^a^	0.12 ± 0.00 ^a^	0.13 ± 0.02 ^a^	0.13 ± 0.03 ^a^	0.21 ± 0.06 ^a^	0.39 ± 0.07 ^b^	0.51 ± 0.01 ^b^
**Total ID**	**100.00**	**97.99**	**96.79**	**95.26**	**90.09**	**82.76**	**58.00**

n.d.: not determined; total ID: % of identified compounds. ANOVA analysis was performed, with different letters (a–g) indicating significant differences (*p* < 0.05). CTR: control (non ozonated oil); samples 1, 2, 3, 4, 5, and 6 correspond to 10, 20, 40, 80, 160, and 320 min of ozone treatment, respectively.

## Supplementary Materials

The following are available online at https://www.mdpi.com/1420-3049/25/16/3601/s1, Table S1: Correlation coefficients between all data of fatty acids and peroxide values.
