# Supplementary material for: High Efficacy of Ozonated Oils on the Removal of Biofilms Produced by Methicillin-Resistant Staphylococcus aureus (MRSA) from Infected Diabetic Foot Ulcers
Source: Molecules. 2020 Aug 7;25(16):3601. doi: 10.3390/molecules25163601 (PMC7464232; doi:10.3390/molecules25163601)
Supplement: Supplementary file 1 [file molecules-25-03601-s001.pdf]

molecules

MDPI

Article

High Efficacy of Ozonated Oils on the Removal of Biofilms Produced by Methicillin-Resistant *Staphylococcus aureus* (MRSA) from Infected Diabetic Foot Ulcers

Vanessa Silva [1,2,3,4], Cecília Peirone [5,6,7], Joana S. Amaral [8,9], Rosa Capita [10,11], Carlos Alonso-Calleja [10,11], Jose A. Marques-Magallanes [12], Ângela Martins [13], Águeda Carvalho [6], Luís Maltez [5,14], José Eduardo Pereira [5,14], José Luís Capelo [15,16], Gilberto Igrejas [2,3,4], and Patrícia Poeta [1,4,*]

1 Microbiology and Antibiotic Resistance Team (MicroART), Department of Veterinary Sciences, University of Trás-os-Montes and Alto Douro (UTAD), 5000-801 Vila Real, Portugal; vanessasilva@utad.pt
2 Department of Genetics and Biotechnology, University of Trás-os-Montes and Alto Douro, 5000-801 Vila Real, Portugal; gigrejas@utad.pt
3 Functional Genomics and Proteomics Unit, University of Trás-os-Montes and Alto Douro (UTAD), 5000-801 Vila Real, Portugal
4 Associated Laboratory for Green Chemistry (LAQV-REQUIMTE), University NOVA of Lisboa, Lisboa, 2829-516 Caparica, Portugal
5 Department of Veterinary Sciences, University of Trás-os-Montes and Alto Douro (UTAD), 5000-801 Vila Real, Portugal; cecilia_peirone@yahoo.com.ar (C.P.); lmaltez@utad.pt (L.M.); jeduardo@utad.pt (J.E.P.)
6 Hospital Center of Trás-os-Montes and Alto Douro E.P.E., 5000-185 Vila Real, Portugal; aguedasenacarvalho@gmail.com
7 Centre for the Research and Technology of Agro-Environmental and Biological Sciences (CITAB), UTAD, 5000-801 Vila Real, Portugal
8 Centro de Investigação de Montanha (CIMO), Instituto Politécnico de Bragança, 5300-253 Bragança, Portugal; jamaral@ipb.pt
9 REQUIMTE-LAQV, Department of Chemical Sciences, Pharmacy Faculty, University of Porto, 4050-313 Porto, Portugal
10 Department of Food Hygiene and Technology, Veterinary Faculty, University of León, E-24071 León, Spain; rosa.capita@unileon.es (R.C.); carlos.alonso.calleja@unileon.es (C.A.-C.)
11 Institute of Food Science and Technology, University of León, 24004 León, Spain
12 Critical Care Department, University Hospital Braga, 4710-243 Braga, Portugal; jose.regojo@yahoo.com
13 Department of Zootechnics, University of Trás-os-Montes and Alto Douro (UTAD), 5000-801 Vila Real, Portugal; angela@utad.pt
14 Veterinary and Animal Research Centre, University of Trás-os-Montes and Alto Douro (UTAD), 5000-801 Vila Real, Portugal
15 BIOSCOPE Group, LAQV@REQUIMTE, Chemistry Department, Faculty of Science and Technology, NOVA University of Lisbon, 2825-466 Almada, Portugal; jlcm@fct.unl.pt
16 Proteomass Scientific Society, 2825-466 Costa de Caparica, Portugal
* Correspondence: ppoeta@utad.pt; Phone: (+351)-259350466; FAX: (+351)-259350629

Table S1. Correlation coefficients between all data of fatty acids and peroxide values.

	**C14:0**	**C16:0**	**C16:1**	**C17:0**	**C17:1**	**C18:0**	**C18:1n9c**	**C18:1n7**	**C18:2n6c**	**C20:0**	**C20:1**	**C18:3n3**	**C22:0**	**C24:0**	**IP**
**C14:0**		0,90	-0,24	0,93	0,99	0,84	-0,95	-0,97	-0,87	0,43	-0,49	-0,92	0,72	0,80	0,88
**C16:0**	***		-0,52	0,99	0,87	0,99	-0,99	-0,97	-0,99	0,69	-0,79	-0,96	0,95	0,96	0,96
**C16:1**	ns	*		-0,49	-0,16	-0,58	0,45	0,42	0,58	-0,46	0,77	0,43	-0,70	-0,50	-0,56
**C17:0**	***	***	ns		0,90	0,98	-1,00	-0,99	-0,99	0,64	-0,76	-0,97	0,92	0,94	0,96
**C17:1**	***	***	ns	***		0,80	-0,92	-0,95	-0,83	0,37	-0,43	-0,90	0,67	0,76	0,85
**C18:0**	***	***	*	***	***		-0,96	-0,93	-0,99	0,74	-0,86	-0,94	0,98	0,97	0,94
**C18:1n9c**	***	***	ns	***	***	***		0,99	0,98	-0,62	0,74	0,98	-0,90	-0,93	-0,96
**C18:1n7**	***	***	ns	***	***	***	***		0,96	-0,55	0,68	0,97	-0,85	-0,89	-0,95
**C18:2n6c**	***	***	*	***	***	***	***	***		-0,70	0,85	0,96	-0,96	-0,96	-0,96
**C20:0**	ns	**	ns	**	ns	**	*	*	**		-0,72	-0,59	0,78	0,81	0,66
**C20:1**	ns	***	***	**	ns	***	**	**	***	**		0,74	-0,92	-0,81	-0,76
**C18:3n3**	***	***	ns	***	***	***	***	***	***	*	**		-0,87	-0,90	-0,91
**C22:0**	**	***	**	***	**	***	***	***	***	***	***	***		0,95	0,90
**C24:0**	***	ns	***	***	***	***	***	***	***	***	***	***	***		0,88
**IP**	***	***	*	***	***	***	***	***	***	*	***	***	***	***	

$p > 0.05$ not significant (ns); 0,01< p <0.05 *; 0.001< p <0.01 **; $p < 0.001$ ***. There is no statistically significant correlation ($p > 0.05$) between C14: 0 and C16: 1; with C20: 0 and C20: 1.There is no statistically significant correlation ($p > 0.05$) between C16: 0 and C24: 0.There is no statistically significant correlation ($p > 0.05$) between C16: 1 and C17: 0 .; C17: 1; C18: 1n9c; C18: 1n7; C20: 0 and C18: 3n3.There is no statistically significant correlation ($p > 0.05$) between C17: 1 with C20: 0 and C20: 1.The association between C14: 0 is positive and high with C16: 0, C17: 0, C18: 0, C22: 0, C24: 0 and IP. With the remaining fatty acids it is negative (as one increases, the other decreases).
